# Cytotoxicity and radiosensitising activity of synthesized dinitrophenyl derivatives of 5-fluorouracil

**DOI:** 10.1186/1560-8115-20-3

**Published:** 2012-07-19

**Authors:** Khosrou Abdi, Ali Khalaj, Syeed-Naser Ostad, Mohammad Reza Khoshayand

**Affiliations:** 1 Department of Medicinal Chemistry, Tehran, Iran; 2 Department of Nuclear Pharmacy, Tehran, Iran; 3 Department of Pharmacology & toxicology, Tehran, Iran; 4 Department of Drug and Food Control, Faculty of Pharmacy, Tehran University of Medical Sciences, Tehran, Iran

**Keywords:** Nitroaromatic compounds, HT-29 cell line, Radiosensitizing activity, Cytotoxicity, 5-FU

## Abstract

**Background and the purpose of the study:**

Dual functional agents in which nitroaromatic or nitroheterocyclic compounds are attached through a linker unit to mustards and aziridines have shown higher cytotoxicities than the corresponding counterparts to both aerobic and hypoxic cells and enhanced radiosensitizing activity. In the present investigation cytotoxicity and radiosensitizing activity of 2,4-dinitrobenzyl, 2,4-dinitrobenzoyl, and 2,4-dinitrophenacetyl derivatives of 5-fluorouracil which was assumed to release cytotoxic active quinone methidide and 5-fluorouracil under hypoxic conditions on HT-29 cell line under both aerobic and hypoxic conditions was investigated.

**Methods:**

5-fluorouracil derivative X-XIII were prepared by the reaction of the corresponding di-nitro substituted benzyl, benzoyl and phenacetyl halides with 5-fluorouracil protected at N-1 with di-*t*-butoxydicarbonate (BOC) in dimethyl formamide (DMF) in the presence of the potassium carbonate followed by hydrolysis of the blocking group by potassium carbonate in methanol. Cytotoxicity of fluorouracil VIII and tested compounds X-XIII against HT-29 cell line under both aerobic and hypoxic conditions after 48 hrs incubation were measured by determination of the percent of the survival cells using 3-(4,5-Dimethylthiazol-2-yl)-2,5-diphenyltetrazolium bromide (MTT) assay and percent of the dead cells using propidium iodide(PI)-digitonine assay and results were used to calculate the corresponding IC_50_ values. Radiosensitization experiments were carried out by irradiation of the incubations with a ^60^Co source and clonogenic assay was performed to determine the cell viabilities following treatment with the tested compounds and/or radiation. Sensitization Enhancement Ratio (SER) of each tested compound was obtained from the radiation survival curves in the absence and presence of each sensitizer for 37% survival respectively.

**Results and major conclusion:**

Findings of the present study showed that alkylation or acylation of 5-fluorouracil result in compounds which have little or no cytotoxicity and radiosensitizing activity under aerobic conditions, but have high cytotoxicity and radiosensitizing effects under hypoxic conditions. Furthermore radiosensitizing activities of compounds under hypoxic conditions increased by increase in their concentrations and SER of the tested 5-FU derivatives at concentrations higher than 50 μmol were equal or higher than 1.6 which is the minimum effective SER of a radiosensitizer in an *in vitro* assay.

## Introduction

One of the most important reasoning for the resistance of solid tumors to chemotherapy and radiotherapy is the presence of hypoxic cells in these tumors due to insufficient blood supply. Oxygen as a radiosensitizer increases the effects of radiation by the formation of free radicals that damage DNA [1]. Nitroaromatic and nitroheterocyclic compounds known as electron affinic compounds act like oxygen in the hypoxic cells and increase the sensitivity of these cells to radiation by formation of radical anions through one electron reduction which is reversed by oxygen in aerobic cells [2]. In the absence of radiation, radiosensitizers are also toxic to hypoxic cells due to the formation of the nitro radical anion and suppressing intracellular sulfhydryl compounds which are radioprotectors [3]. In the normal oxygenated cells in which the initially reduced species can be readily re-oxidized by molecular oxygen, the cytotoxicity of the radiosensitizers has been attributed to the parent nitro compound or its bioactivation by NADPH:quinine oxidoreductase which can act as an oxygen insensitive reductase [4].

Several hybrid or dual functional agents have been described in which nitroaromatic or nitroheterocyclic compounds are attached through a linker unit to cytotoxic nitrogen mustards [2] and aziridine moieties [5] which act as a leaving group. These compounds have shown higher cytotoxicities and enhanced radiosensitizing activity on both aerobic and hypoxic cells than the corresponding counterparts [5]. In these compounds cellular reduction of the nitro group to more electron donating species activate the nitrogen of the cytotoxic group by the release of electron through the aromatic ring and generate reactive alkylating species[2]. Previously it was shown that 3[3-(2,4-dinitro-phenylamino)-propyl]-5-fluoro-1 H-pyrimidine-2,4-dione resulting from linkage of 2,4-dinitrophenylamine through three carbon atoms with 5-fluorouracil which is not a leaving group, under aerobic conditions is not cytotoxic but is radiosensitizer [6].

**Scheme 1:**
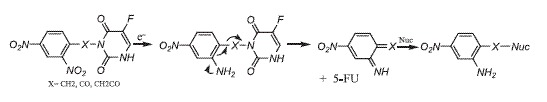
Proposed metabolic reduction of compounds X-XIII on the basis of reference 7.

In the present study, the cytotoxicity and radiosensitizing activity of 2,4-dinitrobenzoyl, 2,4- and 3,5-dinitrobenzoyl and 2,4-dinitrophenylacetyl derivatives of 5-fluorouracil which was assumed to produce quinone methides and 5-fluorouracil [7] by increase in the electron density of the aromatic ring through reduction of the nitro to electron releasing groups under both aerobic and anaerobic conditions was investigated (Scheme 1).

## Material and methods

### Materials and instrumentation

5-Flourouracil, 2,4-dinitrobenzoic acid, 2,4-dinitrophenylacetic acid, 3,5-dinitrobenzoyl chloride, 2,4-dinitrobenzyl chloride, di-t-butoxydicarbonate (Boc)_2_O, thionyl chloride, 4-Dimethyl aminopyridine (4-DMAP), digitonine, and other chemicals and solvents not listed here were from Merck Chemical Co. (Germany). HT-29 Cells (Human colon cancer cells) from Pasteur Institute (Iran), RPMI 1640 from Gibco (England), Fetal Bovine Serum (FBS), Penicillin/streptomycin 100X, Trypsin-EDTA 10X and Trypan blue from Biosera (England),MTT and PI from Sigma (USA) were used in this study. Thin layer chromatography was carried out using silica gel (Kieselgel 60, 230–400 mesh, Merck) to monitor the progress of reactions. Melting points were determined on a MEL-270 Sibata melting point apparatus and are uncorrected. ^1^HNMR spectra were recorded on 500 MHz Bruker (Germany), using DMSO-d_6_ or CDCl_3_ as solvent. Chemical shifts (δ) are reported in ppm relative to TMS as internal standard. Mass spectra were obtained on a Finningan TSQ-70 instrument. Infrared spectra were recorded on a Nicolet Magna IR- 550 spectrometer (USA). All compounds were characterized by the analytical and spectral data. 3,5-dinitrobenzoyl chloride and 2,4-dinitrobenzyl chloride were commercially available and the known 2,4- dinitrobenzoyl, and 2,4-dinitrophenylacetyl chloride were prepared by the reported method [8] through the reaction of the corresponding acids with thionyl chloride. Analytical and spectroscopic data for the known compounds were consistent with the reported literature values and data only for new compounds are presented.

### Chemistry

#### Preparation of compounds X-XIII

Synthesis of 3-(2,4-Dinitrobenzyl)-5-fluoropyrimidine-2,4(1 H,3 H)-dione X:

A solution of 5-Fu-Boc IX (2.30 g, 10 mmol) prepared by the reported method [9] in 30 ml of DMF, was treated with K_2_CO_3_ (1.38 g, 10 mmol), KI (0.41 g, 2.5 mmol), and 2,4-dinitrobezyl chloride (2.2 g, 10 mmol) and the mixture was heated at 78°C for 4 hrs. The mixture was then treated with water, acidified with HCl, and extracted with ethyl acetate (3 × 50 ml) and the organic layer was washed with cold water (5 × 30 ml). The organic phase was evaporated under reduced pressure and the residue was dissolved in MeOH (30 ml) and treated with K_2_CO_3_ (1.4 g, 5.1 mmol) and the reaction mixture was stirred for 2 hrs at room temperature. After evaporation of methanol under vacuum, the residue was diluted with EtOAc (30 ml) and washed with cold water (30 ml). The organic phase was dried over Na_2_SO_4_, evaporated under reduce pressure and the residue was purified by flash column chromatography (silica gel, hexane-EtOAc, 70:30) to give 1.74 g (56%) of the compound X as pale yellowish white color solid; mp: 252-254°C (EtOAc, hexane); IR (KBr) : 3129, 1736, 1659, 1541, 1347 cm^-1^; ^1^ H NMR (500 MHz, DMSO-d_6_): 5.33 (s, 2 H, CH2), 7.63 (d, *J* = 8.5 Hz, 1 H, H6 phenyl), 8.01. (d, *J* = 8.5,1 H, H-6 FU), 8.43 (dd, *J* = 8.5, 2.5 Hz, 1 H, H5 phenyl), 8.79 (d, *J* = 2.5 Hz 1 H, H3 phenyl); MS: 310 (7%), 264(43%), 181(100%), 167 (75%), 130 (70%), 91 (44%), 75 (76%).

Synthesis of 3-(2,4-dinitrobenzoyl)-5-fluoropyrimidine-2,4(1H,3H)-dione XI, 3-(2-(2,4-dinitrophenyl)acetyl)-5-fluoropyrimidine-2,4(1H,3H)-dione XII and 3-(3,5-dinitrobenzoyl)-5-fluoropyrimidine-2,4(1H,3H)-dione XIII:

A mixture of 5-Fu-Boc IX (2.30 g, 10 mmol) [9] and K_2_CO_3_ (1.3 g, 10 mmol) in 20 ml of CH_3_CN were stirred for 30 min and the mixture then was added dropwise over 30 min to a well-stirred, ice-cold CH_3_CN (25 ml) solution containing 10 mmol of the appropriate dinitrobenzene acid chloride. The mixture was stirred for another one hour at 0°C, filtered and washed with CH_3_CN (30 ml). After evaporation of the solvent under reduced pressure, the residue was crystallized from an appropriate solvent to give pure compounds XI-XIII.

XI: As pale yellowish white color (73%); mp: 195-198°C (EtOAc, hexane); IR (KBr): 3131, 1734, 1656, 1536, 1349 cm^-1^; ^1^ H NMR (500 MHz, DMSO-d_6_): 8.01 (d, *J* = 8.5 Hz, 1 H, H-6 FU), 8.69 (d, *J* = 6.5 Hz, 1 H, H6 phenyl), 8.77 (dd, *J* = 6.5, 2.5 Hz, 1 H, H5 phenyl), 8.91 (d, *J* = 2.5 Hz 1 H, H3 phenyl); MS : 324 (5%), 278 (20%), 277 (45%), 195(100%), 167 (26%), 130 (72%), 91 (42%), 75 (78%).

XII: As pale yellowish white color solid (57%), mp: 222-225°C (EtOAc, hexane); IR (KBr): 3125, 1730, 1652, 1540, 1345 cm^-1^; 1 H NMR (500 MHz, DMSO-d_6_): 3.35 (s, 2 H, CH2), 8.03 (d, *J* = 8.5 Hz, 1 H, H6 phenyl), 8.69 (d, *J* = 6.5, 1 H, H-6 FU), 8.77 (dd, *J* = 8.5, 2.5 Hz, 1 H, H5 phenyl), 8.91 (d, *J* = 2.5 Hz 1 H, H3 phenyl); MS: 338 (8%), 292 (18%), 209 (100%), 181 (19%), 157(38%), 130 (70%), 91 (47%), 75 (74%).

XIII As pale yellowish white color solid, (67%), mp: 184-187°C (EtOAc, hexane); IR (KBr): 3133, 1738, 1656, 1547, 1350 cm^-1^; ^1^ H NMR (500 MHz, DMSO-d_6_): 7.76 (d, *J* = 6.5 Hz, 1 H, H-6 FU), 9.15 (d, *J* = 3.1 Hz, 2 H, H2, H6 phenyl), 9.25 (d, *J* = 3.1 Hz 1 H, H4 phenyl); MS : 324 (6%), 278 (23%), 277 (40%), 195(100%), 167 (28%), 130 (70%), 91 (43%), 75 (75%).

## Biology

### Cell culture

Studies were carried out with HT-29 cells, originally derived from human colorectal carcinoma, obtained from Pasteur Institute of Iran. Cells were grown as an attached monolayer in RPMI 1640 supplemented with 10% fetal bovine serum, penicillin (50 unit/ml), and streptomycin (50 μg/ml). Cells were routinely grown in tissue culture flasks and kept in a humidified atmosphere of 5% CO_2_ and 95% air at 37°C.

### Determination of the cytotoxicity

The experiments were carried out in 96-well culture dishes. HT-29 cells were seeded at the density of 10^4^ cells/well. Compounds VIII and X- XIII were dissolved in DMSO to a stock concentration of 50 mmol and stored at -20°C. The stock solutions were diluted with culture media to final concentrations of 6.75-400 μmol and added to each well of dish. Culture mediums containing DMSO in concentration equal to those which were used in incubations treated with the tested compound served as control. Cell incubations were kept at 37°C for 48 hrs under air by purging with 95% nitrogen and 5% CO_2_ or hypoxia in sealed GasPak jar by using Anoximat (Netherland) and an anaerobic chamber GasPak Jar (Merck, Germany) and GasPak pluss (Merck, Germany) [10]. After removal of the supernatant of the culture medium, wells were washed with phosphate buffer saline (PBS), and the numbers of live or dead cells were determined by MTT [11] and PI [12] assays respectively as follows:

### MTT assay

To each well of a 96 well plate was added 25 μl of MTT (5 mg/ml in PBS) and incubated for 4 hrs at 37°C and then 100 μl of DMSO was added to dissolve the precipitated formazan and its absorbance was read in Elisa reader at 550 nm and a background wavelength of 690 nm.

Survival was scored by the ratio of the absorbance of the treated cells to untreated cells with the tested compounds (control) and is expressed as percentage of the cell survival. Cytotoxicity was assessed using cell survival curves in which the percentages of cells that survive were plotted as a function of concentration of tested compounds from which the corresponding IC_50_ values were determined [11].

### PI-digitonine assay

To each well of a 96 well plate was added 20 μl of a 1.53 mmol solution of propidium iodide (PI) in PBS for a final concentration of 30 μmol. After 60 min incubation at 37°C the initial fluorescence intensity from the dead cells was measured in a multi-well plate reader, (BioTek, Synergy HT, USA) with 530-nm excitation and 645-nm emission filters. Then digitonine (600 nmol) was added to each well to permeabilize all cells and label all nuclei with PI and after 30 min incubation, the fluorescence intensity was measured again to obtain a value corresponding to the total number of cells [12]. The percentage of dead cells was calculated as the proportion of the initial fluorescence intensity over that corresponding to the total number of cells [13]. Cytotoxicity was assessed using cell survival curves in which the percentages of cells that survive were plotted as a function of the concentration of the tested compounds from which the corresponding IC_50_ values were determined.

### Radiosensitization experiments

Cell cultures were exposed to a range of concentrations (0, 25, 50, 100 and 200 μmol) of each compound which were lower than their corresponding IC_50_ values and incubated in air or hypoxic condition at 37°C for 3 hrs. Incubations were then irradiated with 5 different doses of radiation (2, 4, 6, 8 and 12 Gy) at room temperature at the dose rate of 1.67 Gy/min and Source Surface Distance (SSD) = 80 cm^2^ by a ^60^Co source (Theratron 780 MDS – Nordion).

### Clonogenic forming assay and cell survival curves

The medium of the cell cultures after treatment with the tested compounds and/or irradiation were first separated to remove the tested compounds, and washed with PBS. Cells were seeded in 6-well plates at concentration of 5 × 10^3^ cells per well and maintained at 37°C for 14 days. The cells were then fixed in methanol, colonies which were formed stained with crystal violet and those containing more than 50 cells were scored. Sensitization Enhancement Ratio (SER) were obtained from the survival curves of irradiation in the presence and absence of the tested compounds from the equation SER = D_0_ untreated cells/D_0_ treated cells, where D_0_ values represent radiation dose that leading to 37% cell survival [14].

### Statistical analyses

For the cell survival and cell death assays each experiment was repeated three times and results are mean ± SD of three independent experiments_._ Statistical analysis was performed using SPSS11.0 for window (SPSS Inc, USA) and descriptive statistics are shown as arithmetic mean ± standard deviation. Independent samples’ *t*-test was used to investigate the differences between irradiated and unirradiated cells treated with the tested compounds and for comparing more than two groups One Way Analysis of Variance were performed and p value smaller than 0.05 was considered statistically significant.

## Results and discussion

Direct acylation or alkylation of 5-fluorouracil usually give a mixture of N-3, N-1, and N-1,3 substituted compounds [15] and preparation of the N-3 derivatives by this approach result in low yield and require chromatographic separation. Therefore three-step sequences involving protection of the N-1 position, acylation or alkylation at N-3 and removal of the protecting group have been employed for the preparation of N-3 substituted 5-fluorouracils.

**Scheme 2:**
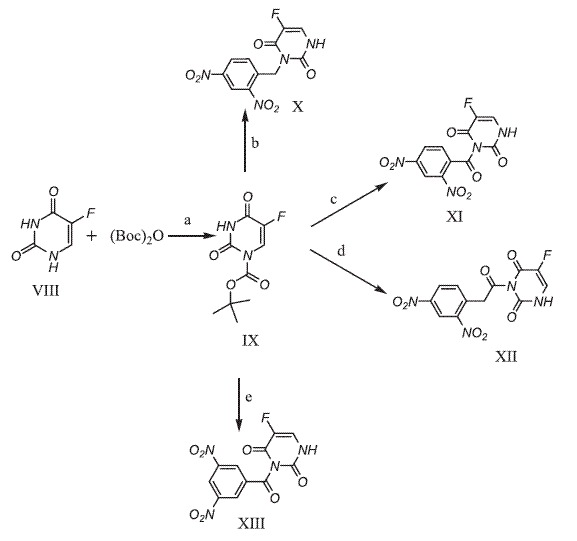
**(a) 4-DMAP/CH**_**3**_**CN (b) 2,4-dinitrobenzyl chloride, K**_**2**_**CO**_**3**_**, KI, DMF (c) 2,4-dinitrobenzoyl chloride, K**_**2**_**CO**_**3**_**, CH**_**3**_**CN(d) 2,4-dinitrophenylacetyl chloride, K**_**2**_**CO**_**3**_**, CH**_**3**_**CN (e) 3,5-dinitrobenzoyl chloride, K**_**2**_**CO**_**3**_**, CH**_**3**_**CN.**

For the protection of N-1 position, the use of acyl [16], benzyl [17], benzyloxymethyl [18], tetrahydropyranyl [19], 2-(trimethylsilyl)ethoxymethyl (SEM) [20] and di-t-butoxydicarbonate [9] groups have been described. However introduction or removal of the most of these groups are associated with some difficulties and in this study BOC which is introduced and removed rapidly and its uses is practical and economical was employed for the protection of N-1 position of 5-FU [9]. Reaction of the resulting N-1 protected 5-fluorouracil with di-nitro substituted benzyl, benzoyl and phenacetyl halides in DMF in the presence of the potassium carbonate resulted in the formation of the corresponding N-3 derivative which upon treatment with potassium carbonate in methanol for removal of the protecting group gave compounds X-XIII (Scheme 2).

**Figure 1:**
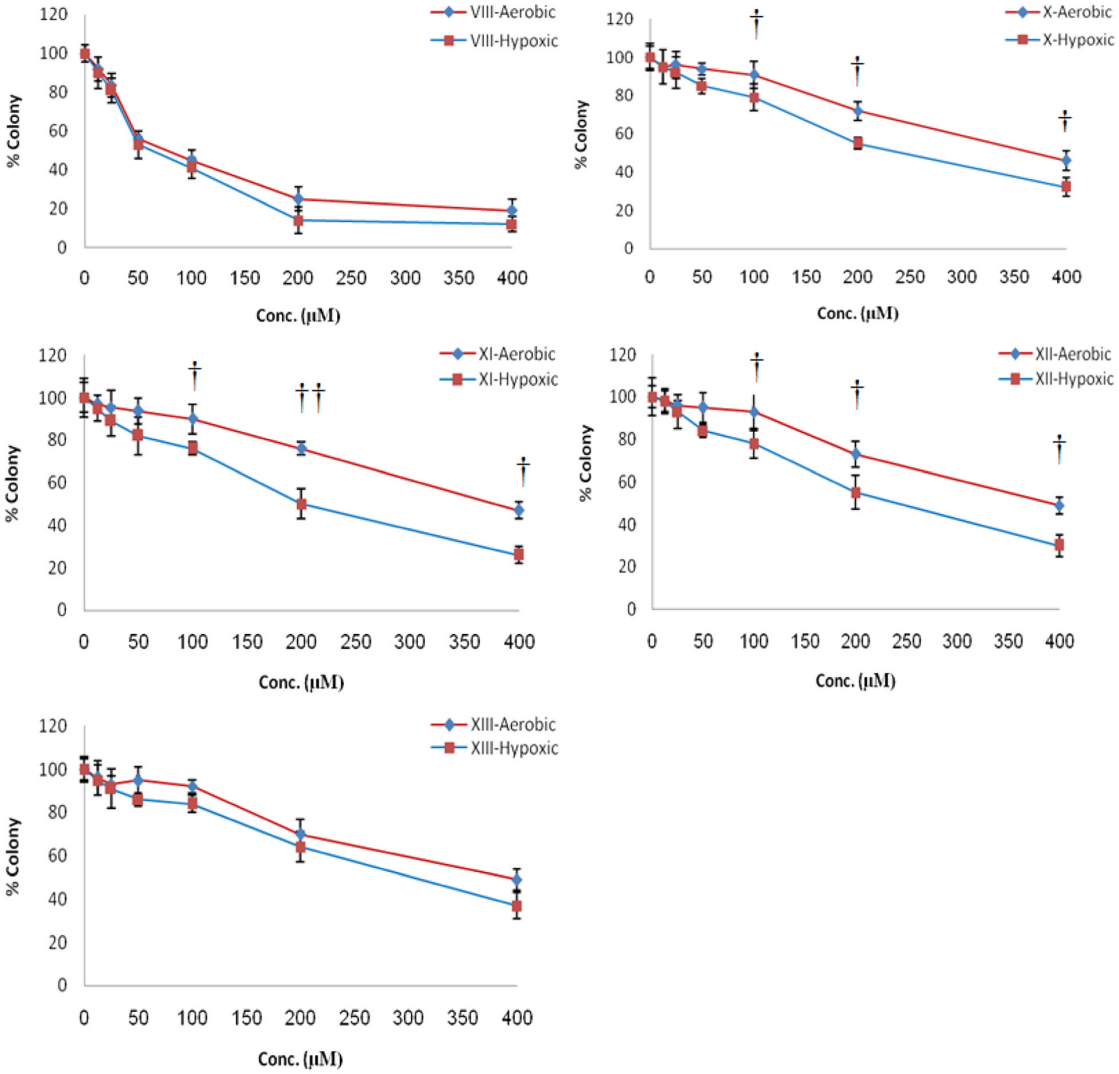
Clonogenic cell survival after 48 hrs incubation of HT-29 cell line with different concentration (6.25, 12.5, 25, 50, 100, 200, 400 μmol) of 5-Fluorouracil VIII and tested compounds X-XIII under Aerobic (♦) and Hypoxic (■) conditions (Mean ± SD), († = P < 0.05, †† = P < 0.01).

Cytotoxicity of 5-Fu and tested compounds X-XIII against HT-29 cell line under both aerobic and hypoxic conditions after 48 hrs incubation, were determined by measurement of the percent of the cell survival using MTT assay [11] and the percent of the dead cell using PI-digitonine assay [12]. The results of these assays were used to obtain the IC_50_ values of the tested compounds as well as the dose of the tested compounds at which under aerobic conditions less than 10% growth inhibition occurred (< IC_10_) as an appropriate dose for the radiosensitization experiments. The cytotoxicity of the compounds under both aerobic and hypoxic conditions were similar after 3 hrs and within this period of time there was no differential cytotoxicity at various concentrations. However after 48 hrs the cytotoxicity of the tested compounds were significantly different. Results of this study showed that IC_50_ values measured by PI-digitonine assay were not significantly different from those determined by MTT assay (Table 1). While the tested compounds under hypoxic conditions at different concentration reduced the cell viability significantly (Figure 1 and Table 1), these compounds were not cytotoxic under aerobic conditions and at concentrations up to 100 μmol reduced the cell viability only to less than 10%.

**Table 1:** **The IC**_**50**_**values for 5-Fluorouracil VIII and tested compounds X-XIII determined by MTT and PI assays on human colon cancer (HT-29) cells under aerobic and hypoxic conditions**

	IC_50_					
		**VIII**	**X**	**XI**	**XII**	**XIII**
^*^MTT	**Aerobic**	**93 ± 9**	**350 ± 15**	**372 ± 19**	**382 ± 12**	**386 ± 24**
	**Hypoxic**	**89 ± 7**	**308.9 ± 19**	**301 ± 16**	**302.1 ± 20**	**350 ± 17**
**PI	**Aerobic**	**88 ± 7**	**345 ± 14**	**380 ± 20**	**393 ± 18**	**379 ± 9**
	**Hypoxic**	**84 ± 5**	**301 ± 19**	**311 ± 12**	**317 ± 17**	**342 ± 21x**

Data points were obtained from three independent experiments, and the standard deviations are given for each point (Mean ± SD).

*: 3-(4,5-Dimethylthiazol-2-yl)-2,5-diphenyltetrazolium bromide.

**: Propidium Iodide.

The cytotoxicity of 5-FU was not significantly different under both aerobic and hypoxic conditions which is in agreement with results of other investigations that N-acylation or alkylation of 5-Fu reduces its cytotoxicity [15].

In contrast to a number of (*O-* and *P*- Nitrobenzyloxycarbonyl)-5-fluorouracils which have shown [7] equal cell killings under both aerobic and hypoxic conditions, which has been attributed to the instability and decomposition of compounds, the cytotoxicity of the tested compounds of this study under hypoxic in comparison with aerobic conditions were higher indicating the possibility that under this condition one electron reduction of these compounds produce cytotoxic reactive intermediates. With exception of 5-FU which under both conditions and compound XIII which under hypoxic conditions showed highest and lowest IC_50_ values respectively, IC_50_ values of other tested compounds under either conditions were not significantly different from each other indicating that they have similar mechanism of action and might produce the same reactive species. The low cytotoxicity of compound XIII could be attributed to the presence of the nitro groups in the *meta* position which in contrast to *ortho* positions after one electron reduction does not produce reactive quinone methiodide species and 5-FU.

Radiosensitization experiments was carried out under conditions identical to the cytotoxicity measurement using MTT assays by incubating HT-29 cells with compounds under hypoxic and aerobic conditions at 37°C. After 3 hrs where none of the compounds had shown significant cytototoxicity, cells were irradiated with a ^60^Co source. Radiosensitivity of the tested compounds were determined through measurement of the cell growth inhibition using colony formation [13] and the clonogenic cells was determined by staining with crystal violet and counting on Dissecting microscope. Results are presented in terms of Sensitization Enhancement Ratio (SER) which were obtained from the survival curves of irradiation in the presence and absence of the tested compounds from the equation SER = D_0_ untreated cells/D_0_ treated cells, where D_0_ values represent radiation dose that leads to 37% cell survival [21].

A comparison of the sensitizer enhancement ratio values of the tested compounds (Table 2) revealed that under aerobic condition none of the compounds and under hypoxia 5-fluorouracil VIII and its 3,5-dinitrophenyl derivative XIII had no radiosensitizing activity.

**Table 2:** Sensitizing Enhancement Ratios (SER) of different concentrations (25, 50, 100, 200 μmol) of 5-Fluorouracil VIII and tested compounds X-XIII on human colon cancer (HT-29) cells upon exposure to different doses of radiation(0, 2, 4, 8 and 12 Gy) under aerobic and hypoxic conditions

Compound	Concentration (μmole)	SER	
		Hypoxic	Aerobic
VIII	25	1.04 ± 0.04	1.09 ± 0.06
	50	1.18 ± 0.11	1.12 ± 0.09
	100	1.20 ± 0.12	1.16 ± 0.10
	200	1.29 ± 0.10	1.21 ± 0.15
X	25	1.20 ± 0.12	1.10 ± 0.10
	50	1.51 ± 0.09	1.16 ± 0.11
	100	1.78 ± 0.15	1.24 ± 0.14
	200	2.23 ± 0.21	1.29 ± 0.12
XI	25	1.30 ± 0.18	1.11 ± 0.08
	50	1.57 ± 0.12	1.21 ± 0.16
	100	1.85 ± 0.16	1.23 ± 0.10
	200	2.39 ± 0.10	1.35 ± 0.17
XII	25	1.16 ± 0.14	1.10 ± 0.09
	50	1.37 ± 0.18	1.15 ± 0.10
	100	1.73 ± 0.11	1.26 ± 0.15
	200	2.28 ± 0.21	1.33 ± 0.13
XIII	25	1.14 ± 0.09	1.07 ± 0.10
	50	1.18 ± 0.14	1.13 ± 0.09
	100	1.22 ± 0.23	1.18 ± 0.14
	200	1.37 ± 0.15	1.25 ± 0.11

Means ± SD, each set of experiments was performed in triplicates.

**Figure 2:**
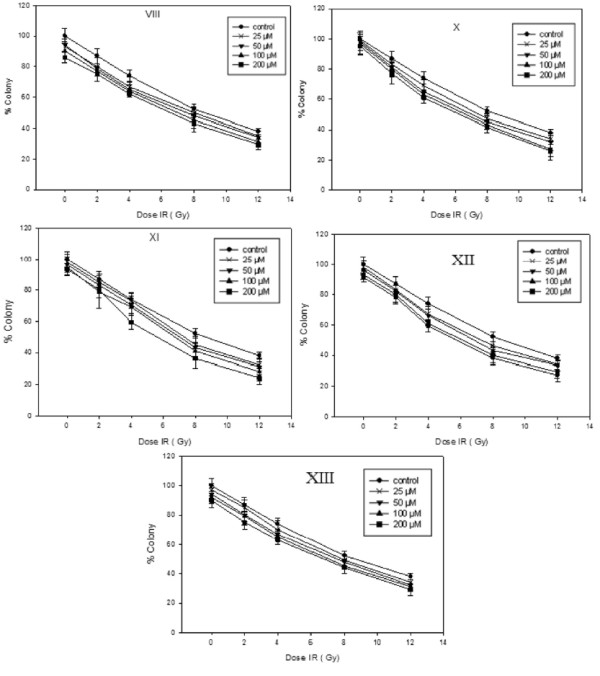
**Clonogenic cell survival of HT-29 cells upon exposure to different dose of Gamma-radiation (2, 4, 6, 8 and 12 Gy) after incubation with different concentration (25, 50, 100 and 200 μmol) of 5-Fluorouracil VIII and tested compounds X-XIII under Aerobic condition.** Data points were obtained from three independent experiments, and the standard deviations are given for each point. (Mean ± SD, each set of experiments was performed in triplicates).

**Figure 3:**
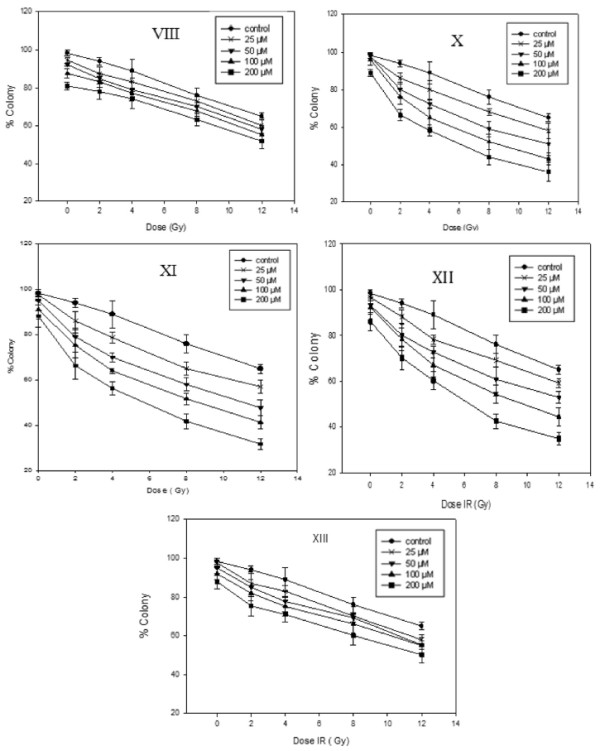
**Clonogenic cell survival of HT-29 cells upon exposure to different dose of Gamma-radiation (2, 4, 6, 8 and 12 Gy) after incubation with different concentration (25, 50, 100 and 200 μmol) of 5-Fluorouracil VIII and tested compounds X-XIII under Hypoxic condition.** Data points were obtained from three independent experiments, and the standard deviations are given for each point. (Mean ± SD, each set of experiments was performed in triplicates).

As it is shown in Figures 2 and 3 cells viability was reduced dose dependently by irradiation which was significant in the presence of the compounds X-XII under hypoxic conditions. Radiosensitizing activities of these compounds increased by increase in their concentrations and at concentrations higher than 50 were equal or higher than 1.6 which is the minimum effective SER [22] of a radiosensitizer in an *in vitro* assay.

## Conclusion

The present study showed that alkylation or acylation of 5-fluorouracil, results in compounds which have little or no cytotoxicity and radiosensitizing activity on aerobic cells but are toxic and radiosensitizer on hypoxic cells and their uses may be advantageous in adjunct cancer treatment by radiotherapy.

## Competing interest

The authors declare that they have no competing interests

## Authors’ contribution

AK: Synthesis and determination of cytotoxicity and radiosensitizing activity of the tested compounds. KA: Designer of the project, supervisor of the synthesis and corresponding author of the manuscript. OS-N: Supervisor for the determination of the cytotoxocity and radiosensitizing activity of the tested compounds. KMR: supervising statistical analyses. All authors read and approved the final manuscript.

## References

[B1] Nagasawa H Uto Y Kirk KL Hori H Design of hypoxia-targeting drugs as new cancer chemotherapeutics Biol Pharm Bull 2006 29 12 2335 42 10.1248/bpb.29.2335 17142959

[B2] Lee HH Palmer BD Wilson WR Denny WA Synthesis and hypoxia-selective cytotoxicity of A 2-nitroimidazole mustard Bioorg Med Chem Lett 1998 8 1741 1744 10.1016/S0960-894X(98)00305-9 9873426

[B3] Tao C Desai NP Soon-Shiong P Sandford PA Dual functional cytotoxic/radiosensitizer compounds and methods for the preparation thereof Chem Abstr 1996 126 131664n PCT Int Appl WO 96 40,091

[B4] Palmer BD Wilson WR Pullen SM Denny WA Hypoxia-selective antitumor agents. 5. Synthesis of water-soluble nitroaniline mustards with selective cytotoxicity for hypoxic mammalian cells J Med Chem 1990 35 3214 3222 1507207 10.1021/jm00095a018

[B5] Ahmed L Jenkins TC Walling JM Stratford IJ Sheldon PW Adams GE Fielden EM Analogues of RSU-1069: radiosensitization and toxicity in vitro and in vivo Int J Radiat Oncol Biol Phys 1986 12 1079 1081 10.1016/0360-3016(86)90230-0 3755711

[B6] Khalaj A Doroudi AR Ostad SN Khoshayand MR Babai M Adibpour N Synthesis, aerobic cytotoxicity, and radiosensitizing activity of novel 2,4-dinitrophenylamine tethered 5-fluorouracil and hydroxyurea Bioorg Med Chem Lett 2006 16 23 6034 6038 10.1016/j.bmcl.2006.08.122 16990003

[B7] Lin TS Wang L Antonini I Cosby LA Shiba DA Kirkpatrick DL Sartorelli AC (o- and p-nitrobenzyloxycarbonyl)-5-fluorouracil derivatives as potential conjugated bioreductive alkylating agents J Med Chem 1986 29 84 89 10.1021/jm00151a014 3941417

[B8] Serebryakov EA Kislitsin PG Semenov VV Zlotin SG Selective synthesis of 1,2-benzisothiazol-3-one-1-oxide nitro derivatives Synthesis 2001 11 1659 1664

[B9] Jaime-Figueroa S Zamilpa A Guzman A Morgans DJ N-3-Alkylation of uracil and derivatives via N-1-Boc protection Synthetic Communications 2001 31 24 3739 3746 10.1081/SCC-100108223

[B10] Mikihiko N Chizuko H Shigeki M Takashi T Caspase-independent necrotic cell death induced by a radiosensitizer, 8-nitrocaffeine Cancer Sci 2004 95 4 361 366 10.1111/j.1349-7006.2004.tb03216.x 15072596 PMC11158938

[B11] Mosmann T Rapid Colorimetric Assay for Cellular Growth and Survival: Application to Proliferation and Cytotoxicity Assays J Immunol Methods 1983 65 55 63 10.1016/0022-1759(83)90303-4 6606682

[B12] Zhang L Mizumoto K Sato N Ogawa T Kusumoto M Niiyama H Tanaka M Quantitative determination of apoptotic death in cultured human pancreatic cancer cells by propodium iodide and digitonin Cancer Lett 1999 142 129 137 10.1016/S0304-3835(99)00107-X 10463768

[B13] Moore A Donahue CJ Bauer KD Mather JP Simultaneous measurement of cell cycle and apoptotic cell death Methods Cell Biol 1998 57 265 78 9648110 10.1016/s0091-679x(08)61584-8

[B14] Franken NP Rodermond HM Stap J Haveman J van Bree C Clonogenic assay of cells in vitro Nat Protoc 2006 1 2315 2319 10.1038/nprot.2006.339 17406473

[B15] Ozaki S Watanabe Y Hoshiko T Nagase T Ogasawara T Furukawa H Hemura A Ishikawa K Mori H Hoshi A Iigo M Tokuzen R 5-Fluorouracil derivatives. X. Synthesis and antitumor activity of α-alkoxyalkyl-5-fluorouracils Chem Pharm Bull 1986 34 1 150 157 10.1248/cpb.34.150 3698125

[B16] Pogolotti ALJr Failla Santi DV A facile synthesis of 3-alkyluracils and thymines J Hetrocycl Chem 1972 9 1423 1428 10.1002/jhet.5570090643

[B17] Ishikawa I Itoh T Takayanagi H Oshima J Kawahara N Mizuno Y Ogura H The Photocycloaddition Reactions of Uridine and Related Compounds with 2, 3-Dimethyl-2-butene Chem Pharm Bull 1991 39 1922 1927 10.1248/cpb.39.1922

[B18] Kundu NG Khatri SG Studies on Uracil Derivatives and Analogs VII. Synthesis of N1-lodomethyl Derivatives of Uracil and Dihydrouracil Synthesis 1985 323 327

[B19] Manfredine S Baraldi PG Bazzanini R Guarneri M Simoni D A new direct glycosylation of pyrimidine, pyrazole, imidazole and purine heterocycles via their N-tetrahydropyranyl (THP) derivatives J Chem Soc Chem Commun 1994 583 584

[B20] Arias L Guzman A Jamie-Figuera S Lopez FJ Morgans DJ Padilla F Perez-Medrano A Quintero C Romero M Sandoval L Direct N3 Alkylation of Uracil and Derivatives via n1-[2-(trimethylsilyl) ethoxymethyl] Protection Synlett 1997 11 1233 1234

[B21] Cariveau MJ Stackhouse M Cui X Tiwari K Waud W Secrist JA Xu B Clofarabine acts as radiosensitizer in vitro and in vivo by interfering with DNA damage response Int J Radiation Oncology Biol Phys 2008 70 1 213 220 10.1016/j.ijrobp.2007.09.012 18037589

[B22] Nakae T Uto Y Tanaka M Shibata H Nakata E Tominaga M Maezawa H Hashomoto T Kirk KL Nagasdawa H Hori H Design, synthesis, and radiosensitizing activities of sugar-hybrid hypoxic cell radiosensitizers Bioorg Med Chem Lett 2008 16 675 682 10.1016/j.bmc.2007.10.035 18029186

